# Proton boron capture therapy: microdosimetry and treatment planning study with boron

**DOI:** 10.3389/fonc.2025.1615241

**Published:** 2025-10-17

**Authors:** Seung Hoon Yoo, Ilya Sedliarou, Jennifer A. MacDiarmid, Himanshu Brahmbhatt, Soorim Han, Kum Bae Kim, Linh T. Tran, Anatoly B. Rosenfeld, Eun Ho Kim, Yen Hwa Lin, Wei Sing Tan, Ying Ying Cheah, Ru Xin Wong, Wen Shen Looi, Shaun Ho, Kwek Boon Han, Paul Yeo, SK Djeng

**Affiliations:** ^1^ Proton Therapy Private Limited Company (PTE LTD), Singapore Advance Medicine, Singapore, Singapore; ^2^ EnGeneIC Pty Ltd, Sydney, NSW, Australia; ^3^ Yonsei Cancer Center, Medicine of Yonsei University, Seoul, Republic of Korea; ^4^ Department of Radiation Oncology, Korea Institute of Radiological and Medical Sciences, Seoul, Republic of Korea; ^5^ Center for Medical Radiation Physics (CMRP), University of Wollongong, Wolongong, NSW, Australia; ^6^ Department of Biochemistry, School of Medicine, Daegu Catholic University, Nam-gu, Daegu, Republic of Korea

**Keywords:** PBCT, RBE, flash, LET, SOI detector

## Abstract

**Background:**

Proton-boron capture therapy (PBCT) has been proposed as a method to enhance the biological effectiveness of proton therapy through the p + ^11^B → 3α nuclear reaction. The resulting alpha particles may increase local radiation quality, but the dosimetric and microdosimetric consequences remain uncertain.

**Methods:**

Lineal energy distributions were measured using a Silicon-On-Insulator (SOI) microdosimeter under 70 MeV and 190 MeV monoenergetic proton beams delivered with pencil beam scanning. Dose-averaged lineal energy ( $\bar{y_{D}}$ ) values were derived from oscilloscope signals calibrated against Geant4 Monte Carlo simulations. Measurements were performed at both entrance and Bragg peak depths, with and without boronophenylalanine (BPA) delivered via EnGeneIC Dream Vector (EDV™). In parallel, a treatment planning study was conducted in Eclipse TPS to assess the impact of localized high-density boron regions on dose distributions under conventional and FLASH-simulated delivery, using both fixed and variable RBE models.

**Results:**

For 70 MeV protons, no significant difference in $\bar{y_{D}}$ was observed between boron-loaded and control conditions. At 190 MeV, a reproducible increase in $\bar{y_{D}}$ was detected at the Bragg peak in the presence of boron (p < 0.01), while no effect was observed at the entrance depth. Treatment planning simulations showed that localized boron density improved dose uniformity within the clinical target volume and reduced discrepancies between fixed and variable RBE dose distributions under FLASH conditions.

**Discussion:**

These findings indicate that PBCT can induce detectable increases in microdosimetric lineal energy under high-energy proton beams, even in the absence of macroscopic dose enhancement. The treatment planning results further highlight the potential of boron-enhanced LET modulation in conjunction with FLASH delivery. Together, the study supports continued investigation of PBCT as a strategy to optimize biological effectiveness in proton therapy, with future work focusing on realistic boron distribution models and integration of dose-rate effects.

## 1 Introduction

Proton boron capture therapy (PBCT) has gained increasing interest as a potential enhancement for proton therapy following its initial proposal (1, 2), leveraging the nuclear reaction between protons and ¹¹B to produce three alpha particles (3α) via the p + ^11^B → 3α reaction. These densely ionizing secondary particles have the potential to increase biological effectiveness at the site of interaction while preserving the spatial precision of proton dose deposition. However, the exact dosimetric and biological contributions of this interaction remain an active investigation.

Several *in vitro* studies have demonstrated the potential therapeutic benefit of PBCT (3–5), whereas others have reported conflicting or inconclusive results (6–8). Additional research has explored related concepts such as neutron capture enhanced particle therapy (NCEPT), which focuses on ^10^B as a neutron absorber (9). The outcomes of PBCT and NCEPT can vary significantly depending on the particle type, energy spectrum, and irradiation geometry (10). A recent independent validation of PBCT (11) reported significant biological effects; however, the mechanisms driving such enhancements are still not fully understood.

A key concept in understanding the potential of PBCT lies in the distinction between linear energy transfer (LET) and lineal energy (y). LET is a macroscopic quantity defined as the average energy deposited by a charged particle per unit path length (typically in keV/μm) and is often used in treatment planning and radiobiological modelling to estimate radiation quality. In contrast, lineal energy is a microdosimetric parameter that represents the energy deposited per unit track length within a microscopic site volume, typically derived from stochastic measurements of individual particle interactions. While both quantities relate to stochastic energy deposition density, lineal energy captures event-by-event fluctuations of energy deposition in small target volumes, making it more relevant to for predicting biological damage on the cellular scale.

In boron neutron capture therapy (BNCT), microdosimetric techniques—such as those using tissue-equivalent proportional counters (TEPCs)—have been used to measure differences in lineal energy distributions between boron-loaded and non-loaded targets (12). Translating this concept to PBCT requires precise and high-resolution tools for capturing localized LET changes resulting from the p + ^11^B reaction.

Silicon-On-Insulator (SOI) microdosimeter offer a promising platform for such microdosimetric investigation. These detectors, developed with three-dimensional sensitive volumes in micron-scale silicon layers, enable the resolution of individual particle tracks with high temporal and spatial fidelity. The MicroPlus probe utilizing SOI microdosimeter and low noise readout front-end electronics, developed at the Centre for Medical Radiation Physics (CMRP), University of Wollongong, is a notable implementation that incorporates a configurable array of micron sized sensitive volumes for real-time measurement of energy deposition patterns (13–15).

Previous studies have shown good agreement between measured dose-averaged lineal energy ( $\bar{y_{D}}$ ) using SOI microdosimeter and Monte Carlo-calculated dose-averaged LET (LET_d_) values (16). However, other studies (17) have found no significant biological effect enhancement attributable to boron or neutron capture when using SOI-based measurements, suggesting that further methodological refinement is needed.

In this study, we aim to clarify whether measurable increases in dose averaged lineal energy can be observed under clinically relevant proton beam conditions when BPA (boronophenylalanine) is introduced, using an SOI microdosimeter. The oscilloscope signal from the detector is directly calibrated against LET profiles simulated via Geant4 Monte Carlo (18, 19) to estimate lineal energy distributions. Both 70 MeV and 190 MeV proton beams are evaluated, with measurements performed at entrance and Bragg peak regions.

In addition to physical measurement, we perform a planning study to assess whether localized increases in physical density (as a surrogate for concentrated boron regions) could influence RBE-weighted dose distributions under conventional and FLASH dose-rate conditions. The combination of microdosimetric measurement and planning analysis is intended to improve our understanding of PBCT feasibility and to guide future integration into LET-aware treatment strategies.

## 2 Method and materials

### 2.1 SOI microdosimeter

**Figure 1:**
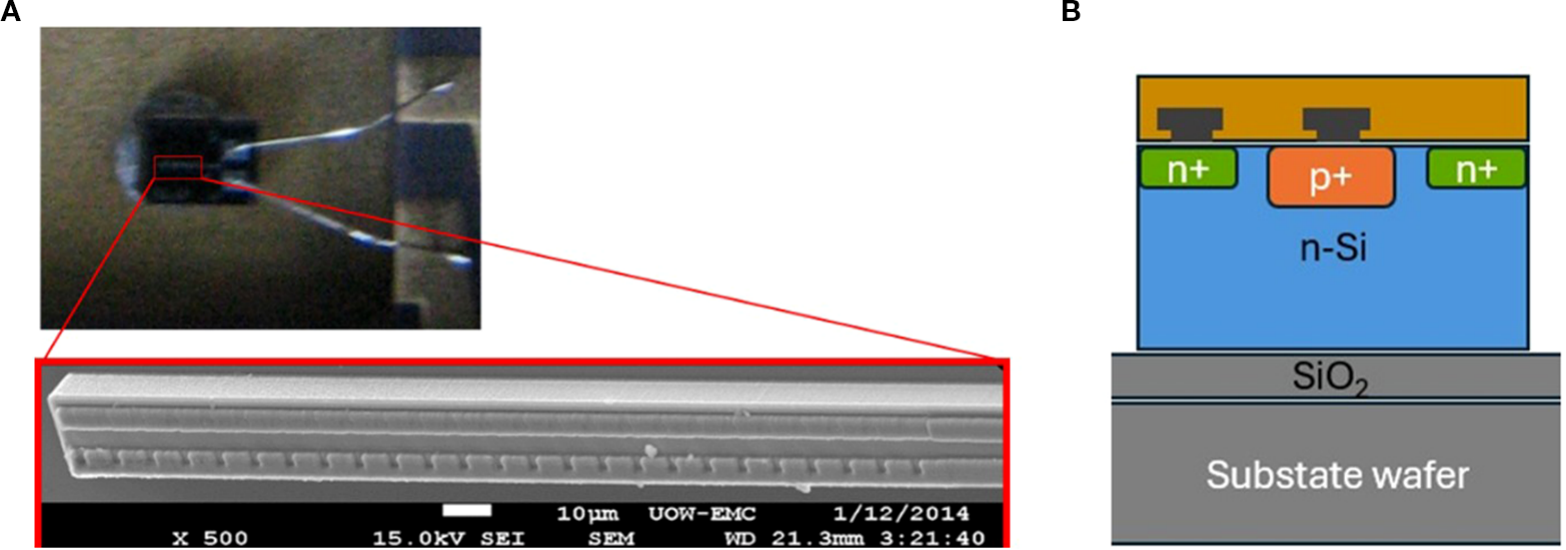
SSMD chip on a DIL package with the inset showing a scanning electron microscope (SEM) image of a single sensitive volume **(A)**, a cross-section diagram of the SSMD detector **(B)**.

A Silicon-On-Insulator (SOI) microdosimeter connected to the readout electronics probe (MicroPlus) was used to measure the lineal energy of proton beams. The SOI microdosimeter is based on the ‘Bridge’ microdosimeter design, which incorporates three-dimensional micro-structured silicon volumes within a 10 µm-thick active layer on an insulating substrate. This geometry enables high spatial and temporal resolution for detecting energy deposition from individual particle interactions. The schematic design of the Bridge microdosimeter is shown in **Figure 1**.

To minimize pile-up effects in high-flux pencil beam scanning (PBS) proton fields, a single sensitive volume microdosimeter (SSMD) was used, featuring a much smaller active area (2,500 µm²) compared to the full bridge array (864,000 µm²). The microscope images and schematic design of the SSMD is shown in **Figure 1**. This reduced active area significantly lowers the probability of pile up, making it suitable for dynamic scanning beam applications.

Details of the microdosimeter design, including the bridge microdosimeter structure and the underlying fabrication process, have been described in previous studies (13–15).

### 2.2 Proton beam irradiation and boron phantom setup

Lineal energy measurements were performed using monoenergetic proton beams of 70 MeV and 190 MeV generated by a Varian ProBeam cyclotron-based system at Proton Therapy PTE LTD, Singapore. A pencil beam scanning (PBS) delivery mode was used with single-energy spot beams to allow precise control of proton energy and depth. The SOI microdosimeter was positioned at isocenter using room lasers, and measurement depth was achieved by stacking RW3 solid water phantoms (PTW) in 1mm increments above the detector. The water-equivalent thicknesses corresponding to the entrance and Bragg peak regions were approximately 2cm and 3.8cm for 70 MeV, and 5cm and 22.5cm for 190 MeV, respectively.

Irradiation was conducted in machine quality assurance (QA) mode with 500,000 monitor units (MU) delivered at each depth. The cyclotron current was adjusted depending on beam energy—400 nA for 70 MeV and 23 nA for 190 MeV—to account for transmission differences, which resulted in higher fluence for the 190 MeV beam. **Table 1** summarizes the beam parameters.

**Table 1:** Irradiation parameters with spot beam.

Setting	Cyclotron current	MU	Transmission efficiency	Fluence at target (protons/cm^2^)
70 MeV	400 nA	500,000	5-10%	Approx. 1.5 × 10^8^
190 MeV	23 nA	500,000	70-80%	Approx. 5.0 × 10^8^

**Figure 2:**
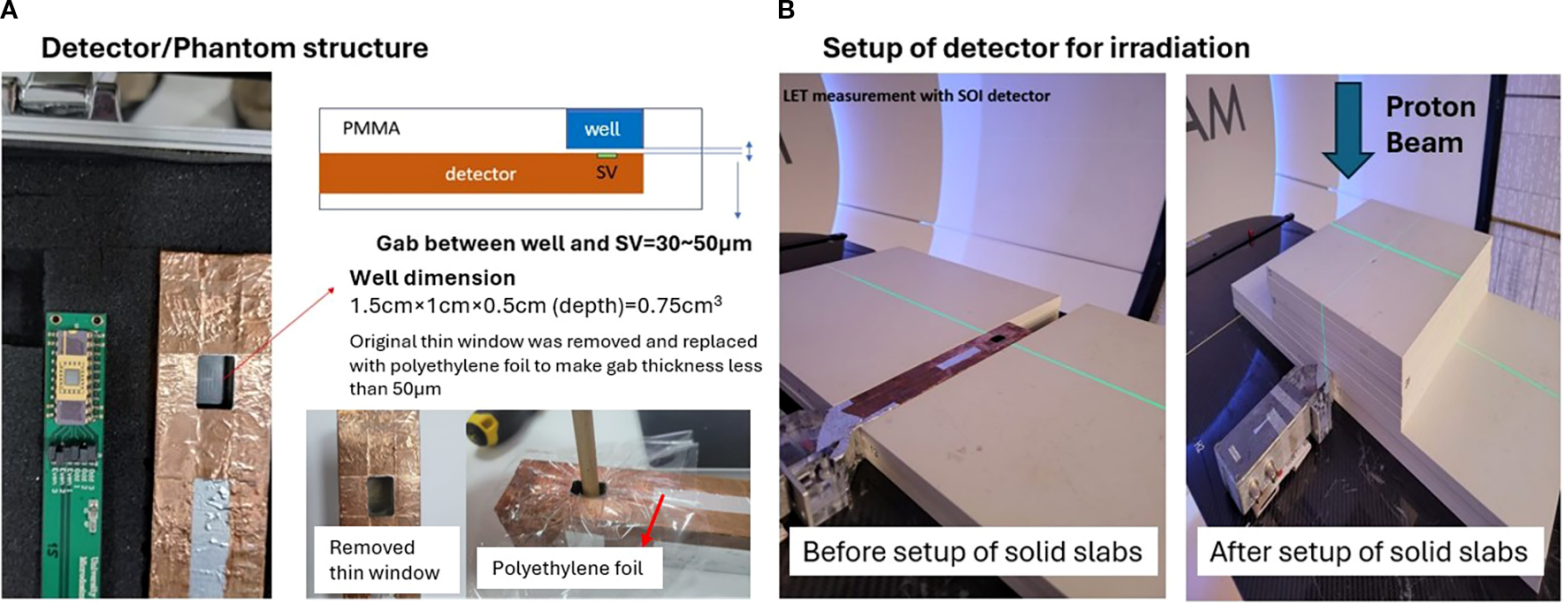
Structure of SOI microdosimeter with PMMA sheath, which window was replaced with Polyethylene foil **(A)** and setup of detector with solid slabs for irradiation **(B)**.

**Figure 2** illustrates the experimental setup used for proton irradiation and SOI microdosimeter positioning. In **Figure 2A**, the schematic shows the structural configuration of the SOI microdosimeter embedded in the PMMA sheath, highlighting the spatial relationship between the detector well and the sensitive volume (SV) through a cross-sectional view. Original PMMA wall of the sheath in window opening was removed, and two folded wrapping plastic was used to insert water and boron with water. This wrapping material, identified as low-density polyethylene (LDPE, density 0.92 g/cm³), had a total physical thickness of approximately 40 μm, which is comparable to ~31 μm of PMMA equivalent thickness. This modification allowed alpha particles generated from the proton–boron reactions to potentially reach the SV, supporting the validity of observed lineal energy changes. **Figure 2B** displays the physical setup before and after positioning the solid PMMA slabs used for depth adjustment and dose modulation in the proton beam path. Water equivalent distance of the solid water slabs was considered for all measurements and analysis.

To evaluate the influence of boron on the lineal energy distribution, a PMMA chamber well (volume: 0.75 cm³) was positioned directly in front of the SOI microdosimeter sensitive volume. The separation gap between the boron-containing well and the sensitive volume ranged from 30 to 50 μm, as measured manually due to fabrication variability. This gap was sufficiently thin to allow alpha particles with energies above approximately 5 MeV—originating from the proton–boron reaction—to reach the detector, enabling partial detection of the high-energy tail of the alpha spectrum. The gap between the boron well and the SV was 30–50 μm PMMA equivalent thickness. Using stopping-power ranges for alphaα particles in PMMA (≈ 33, 45, 58, 73 µm at 5, 6, 7, 8 MeV), alpha particles must have > 5.3 MeV to traverse the gap and reach the SV conservatively. The three-body p + ^11^B → 3α breakup provides a broad alphaα particles’ energy envelope up to the Q-value (8.68 MeV); adopting a conservative, broad distribution, the fraction of alphaα particles above threshold can be about 33% and 22% for 30 µm vs 50 µm gaps respectively.

For vertical irradiation from above at a gantry angle of 0°, RW3 solid water-equivalent slabs were stacked on the treatment couch to achieve the required measurement depths (entrance and Bragg peak) for each proton energy. The slabs were arranged with a depth resolution of 1 mm by varying the number of plates accordingly.

The BPA solution contained natural boron (approximately 80% ^11^B and 20% ^10^B), with a fixed concentration of 100 ppm. EnGeneIC Dream Vector (EDV) (20) was included at 100 ppb as a nanoparticle carrier, consistent with future clinical translation, although its low concentration was negligible in affecting the lineal energy distribution. All solutions were injected using syringes, and the chamber was washed and refilled after each condition to prevent cross-contamination.

BPA was first dissolved in NaOH and subsequently neutralized using phosphate-buffered saline (PBS) prior to mixing with EDV. No stabilizing agents such as fructose or mannitol were used, which are typically part of the standard preparation protocol for *in vitro* or *in vivo* use. While the BPA remained in solution for the duration of the experimental measurements, this approach may have allowed partial precipitation at neutral pH, representing a methodological limitation of this study.

Lineal energy distribution measurements were performed at the entrance and Bragg peak regions for both beam energies, with and without boron. Each setup measurement was repeated at least 10 times to ensure reproducibility and results were statistically evaluated using two-tailed unpaired t-tests.

### 2.3 Lineal energy conversion from oscilloscope signal

$\bar{y_{D}}$ was estimated from the measurements of the lineal energy by directly converting oscilloscope signals recorded from the SOI microdosimeter during proton beam irradiation. Unlike previous studies that employed multi-channel analyzers (MCAs), this study utilized a linear model to convert peak voltage signals into lineal energy values without using an MCA.

Each proton interaction event generated a voltage pulse, with the peak amplitude used as a proxy for energy deposition. These peak values were extracted and binned to generate lineal energy distributions (counts vs. energy). The relationship between the oscilloscope signal and lineal energy was modeled using a linear calibration equation:


(1) Lineal Energy (y)=a×PeakValue+b

Calibration coefficients “a” and “b” were determined for each proton energy (70 MeV and 190 MeV) using Geant4 Monte Carlo simulations with the QGSP_BIC_EMY physics list. The simulation geometry modeled a 10 µm-thick silicon slab representing the SOI microdosimeter sensitive layer. The proton beam source was placed 50cm upstream from the detector, consistent with the beamline geometry used in the experimental configuration. Monoenergetic pencil beams were simulated with a Gaussian spatial distribution (beam sigma = 2mm) and scored for energy deposition per track segment. Lineal energy was computed as the deposited energy divided by the corresponding track length (21, 22). Simulated values were binned using a bin width of 0.5 keV/μm over a range of 0 to 20 keV/μm to generate reference histograms for calibration. The chosen bin width reflected an empirical balance between energy resolution and statistical reliability, considering the measurement sensitivity of the SOI microdosimeter.

The Geant4 simulations employed the QGSP_BIC_EMY physics list with production cuts set to 1 µm for electrons, positrons, and photons, ensuring accurate modelling of electromagnetic interactions relevant to energy deposition in the silicon detector. The hadronic interactions, including elastic and inelastic scattering, were governed by the Binary Cascade model. The geometry included a water-equivalent phantom slab with the 10 µm silicon sensitive layer embedded at the relevant depth corresponding to each measurement point.

Geant4 simulations were performed using the same detector geometry, beam setup, and physics models as the experiment, and were configured to generate reference LET_d_ depth profiles used solely for calibrating the conversion from oscilloscope signal to lineal energy. No direct comparison between simulated LET and measured $\bar{y_{D}}$ was performed.

$\bar{y_{D}}$ was calculated from the measured lineal energy distributions by applying microdosimetric dose-weighting, where the square of the lineal energy is weighted by the frequency of each event. Specifically, $\bar{y_{D}}$ was obtained using the relation $\bar{y_{D}}$ (y) = ∑(y²·f(y))/∑(y·f(y)), where y is the lineal energy and f(y) the corresponding frequency. This approach ensures that the reported values reflect the biologically relevant dose-weighted spectrum rather than the frequency-mean lineal energy.

**Figure 3:**
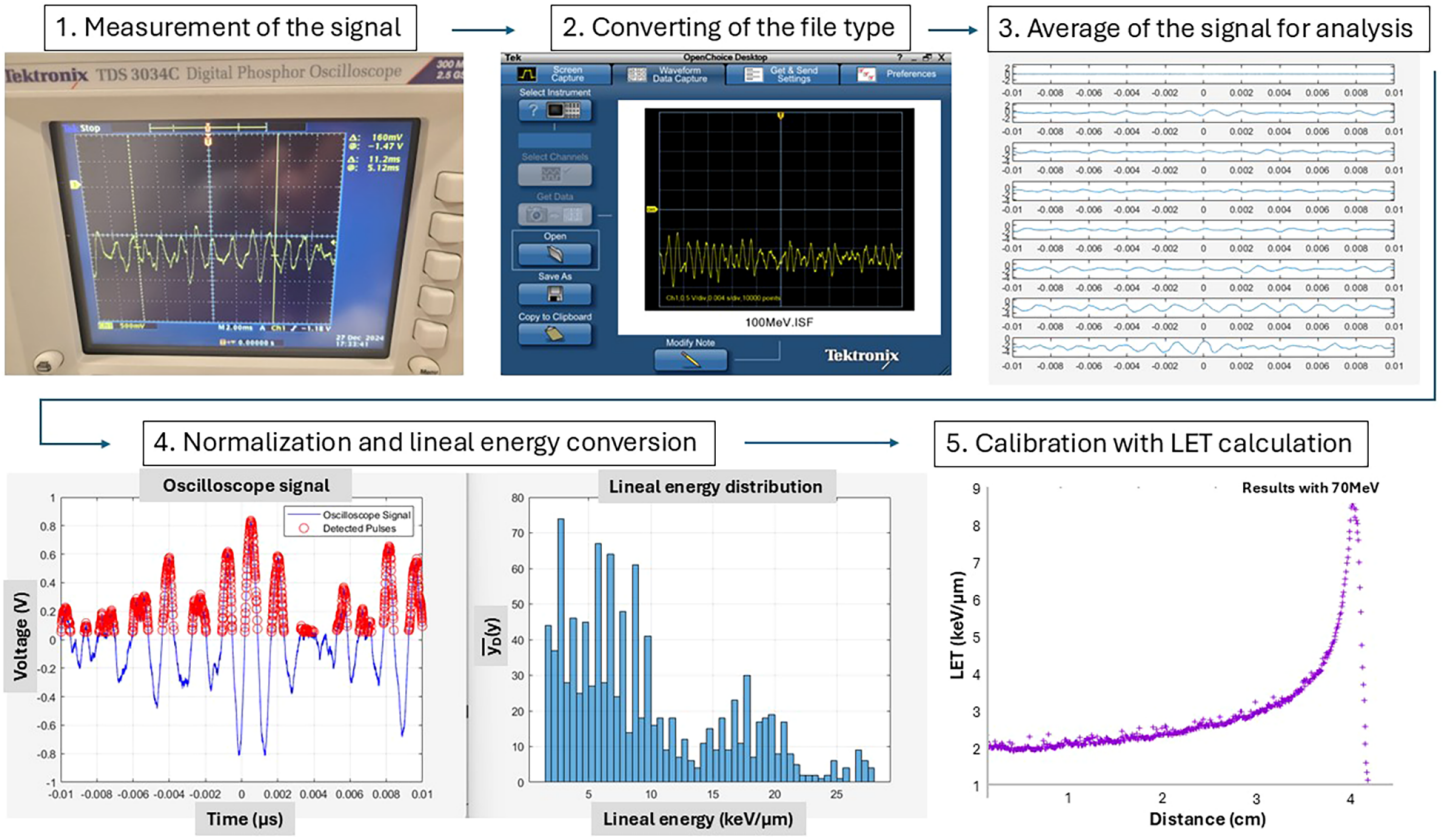
Process of the dose-averaged lineal energy ( $\bar{y_{D}}$ ) conversion from the oscilloscope signal. The flow diagram illustrates the process of extracting peak voltage signals from oscilloscope waveforms. Each detected proton interaction generates a voltage pulse, from which the peak amplitude is extracted to histogram to form the lineal energy distribution. This distribution is calibrated against Geant4 Monte Carlo simulations to convert voltage values to lineal energy using a linear model. Finally, the dose-averaged lineal energy is computed from the distribution.

All measurements were conducted using a 500 mV oscilloscope range with a 2 ms time resolution. To exclude background noise, pre-irradiation signals were used to determine a critical threshold, ensuring that only proton-induced events were analyzed. Post-irradiation, each experimental configuration was measured ten times to calculate average $\bar{y_{D}}$ values and standard deviations. The measurement depths for the entrance and Bragg peak regions were 2cm and 3.8cm for 70 MeV and 5cm and 22.5cm for 190 MeV, respectively. **Figure 3** illustrates the signal processing and $\bar{y_{D}}$ conversion pipeline.

### 2.4 Treatment planning for boron density effect

A dosimetric study was performed using a liver case selected from an anonymized patient dataset at our institution. The liver was chosen due to its clinical relevance in proton therapy, its relatively large target volume, and the uniform tissue composition, which simplify analysis of LET distributions and dose perturbations. Within the clinical target volume (CTV), we introduced a spot-array–like pattern (3mm diameter per spot) as a simulation construct. Each spot was assigned a physical density of 2.34 g/cm³ and a relative stopping power (RSP) of 1.18, chosen not to represent realistic BPA concentrations but to mimic a localized high-density region for evaluating sensitivity of LET-weighted dose calculations in the TPS. Although this is not reflective of typical experimental boron concentrations (100 ppm), this modelling approach explores the theoretical upper-bound impact of boron-enhanced stopping power and LET variation within a small subvolume, which is relevant for targeted boron delivery in future PBCT applications.

Treatment planning was conducted in the Eclipse Treatment Planning System (version 15.6, Varian Medical Systems) using single-field optimization (SFO) with two-field beam arrangement. Dose calculations were performed using both the Proton Convolution Superposition (PCS) algorithm, assuming a fixed RBE of 1.1, and the MicroCalculation script (version 1.5) (23), which incorporates variable RBE modelling based on LET distributions. Two delivery scenarios were evaluated: a conventional dose rate plan using a minimum of 2 MU per spot, and a FLASH-simulated plan using 200 MU per spot. The FLASH simulation was achieved by increasing per-spot dose to deliver higher instantaneous dose rates, exceeding 40 Gy/s during individual spot deliveries based on system beam current and timing characteristics. This approach provides a practical proxy for FLASH delivery conditions, although it does not account for radiobiological effects such as oxygen depletion or radical quenching. The goal of this comparison was to evaluate the impact of boron-enhanced stopping power on dose distributions under different RBE models and delivery rates.

## 3 Results

### 3.1 Depth effect on the $\bar{y_{D}}$

**Figure 4:**
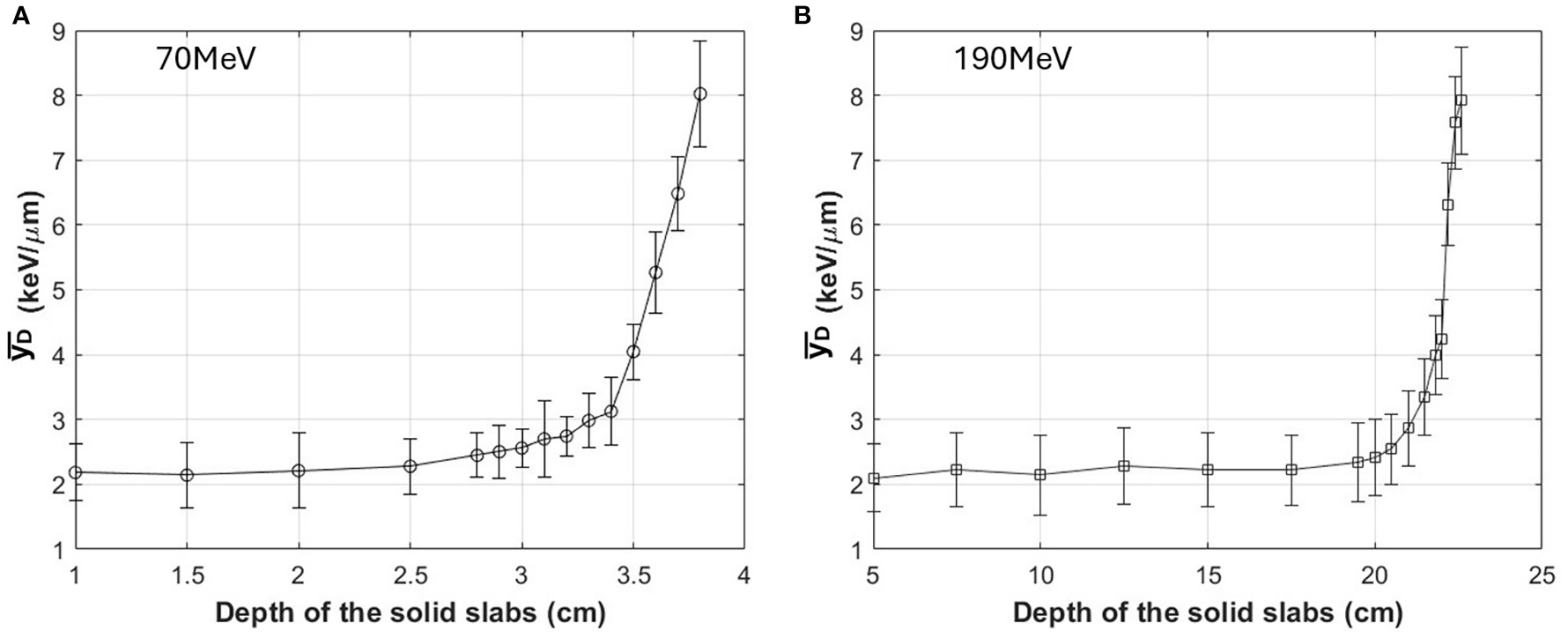
Depth dependence of the $\bar{y_{D}}$ measured with the SOI microdosimeter for 70 MeV **(A)** and 190 MeV **(B)** proton beams, without boron. Measurements were performed at multiple depths from the entrance to the Bragg peak region. The $\bar{y_{D}}$ values were obtained by converting oscilloscope peak voltage signals into lineal energy using calibration coefficients derived from Geant4 Monte Carlo simulations of the detector geometry. Error bars represent the standard deviation from ten repeated measurements at each depth.

**Figure 4** illustrates the variation of $\bar{y_{D}}$ with depth for each beam energy. **Figures 4A, B** show the results for 70 MeV and 190 MeV proton beams, respectively. Measured data were calibrated in the entrance region (2.0cm for 70 MeV and 5cm for 190 MeV) by comparison with Geant4 simulation results.

For the 70 MeV beam, measurements were performed at 0.5cm intervals in the entrance and plateau regions, and at finer intervals of 0.1cm as the measurements approached the Bragg peak. For the 190 MeV beam, measurements were performed at 5cm intervals in the entrance and plateau regions, and at finer intervals of 0.2cm near the Bragg peak. For both 70 and 190 MeV, the highest $\bar{y_{D}}$ value was evaluated around 8 keV/µm, rising from a base line of 2 keV/µm as determined by MC-calibrated values. In the figure, deviation is from the averaged values with 10 times measurements for each depth, with deviations remaining below 0.9 keV/µm.

For each proton energy (70 MeV and 190 MeV), $\bar{y_{D}}$ was calibrated at a single depth point corresponding to the entrance region, using Geant4 Monte Carlo simulations. The calibration parameters (a and b) in the linear conversion equation were derived by matching the simulated and measured lineal energy spectra at this depth. These parameters were then applied consistently to all other depths for the same energy, assuming stable detector response and signal characteristics under the controlled experimental setup. Since proton energy influences both energy deposition patterns and track structure in the detector, separate calibration coefficients were derived for each energy.

### 3.2 Boron effect on the $\bar{y_{D}}$ (spot beam)

**Figure 5:**
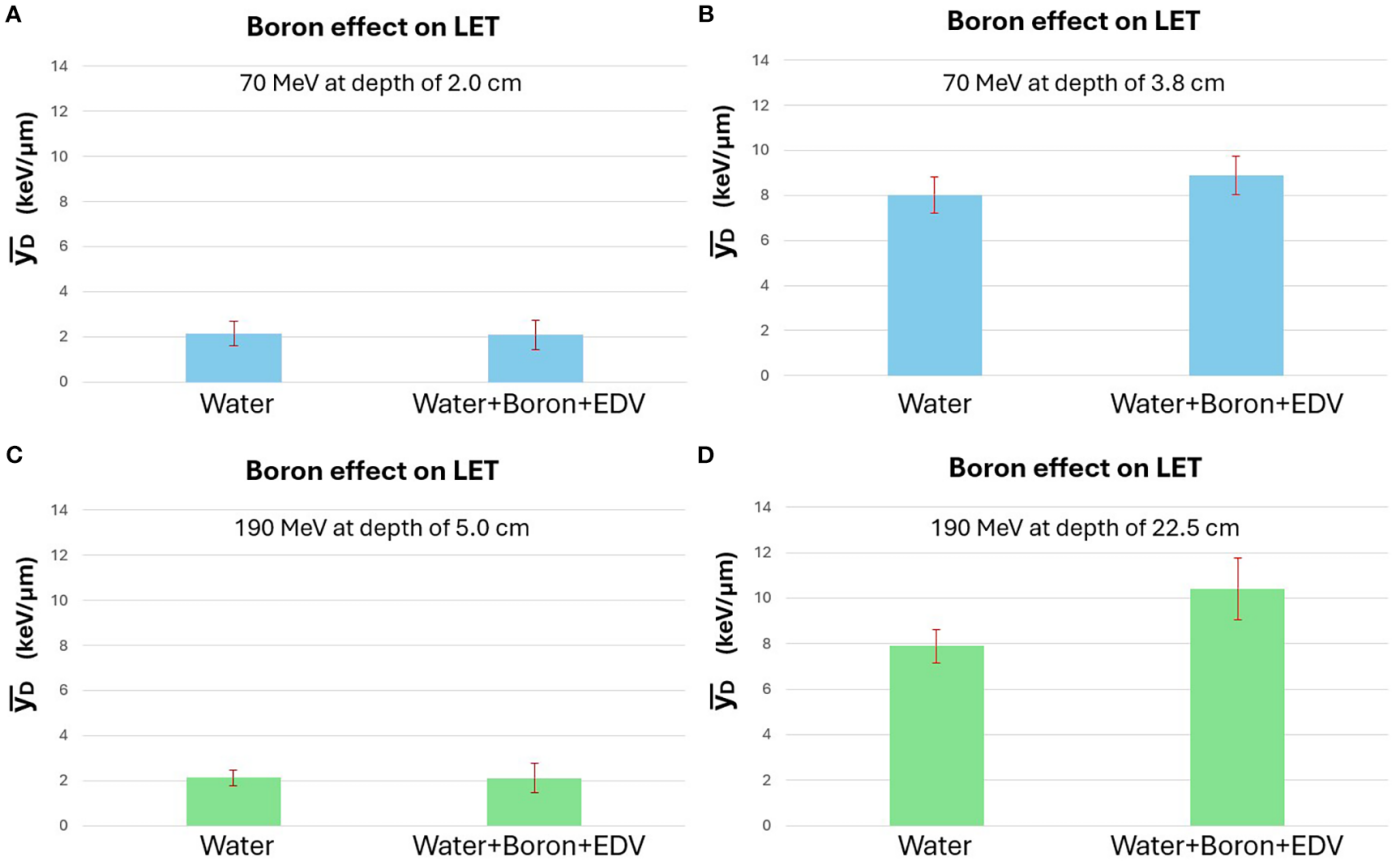
Comparison of $\bar{y_{D}}$ measurements at entrance and Bragg peak depths for 70 MeV and 190 MeV proton beams, with and without BPA (Boronophenylalanine). Boron-containing conditions involved a mixture of water + BPA + EDV (EnGeneIC Dream Vector), with BPA concentration fixed at 100 ppm (boron) and EDV diluted to 100 ppb. **(A, B)** show results for 70 MeV at 2.0cm and 3.8cm depths; **(C, D)** show 190 MeV at 5.0cm and 22.5cm. Statistically significant $\bar{y_{D}}$ increase was observed only for the 190 MeV Bragg peak with BPA (p < 0.01). Error bars indicate standard deviations from 10 repeated measurements.

**Figure 5** presents the effect of boron on $\bar{y_{D}}$ measured at both entrance and Bragg peak depths. In this study, “boron” specifically refers to boronophenylalanine (BPA), which was used as the boron-delivering compound. Three experimental conditions were tested: (1) water only (no BPA, no EDV), (2) water containing BPA combined with EnGeneIC Dream Vector (EDV™) as a carrier and (3) water+EDV only. The BPA concentration was fixed at 100 ppm (in terms of boron content), while EDV was diluted to 100 ppb. The same boron-containing condition was applied in all BPA measurements, and no separate BPA-only condition was tested.

For condition (3) water+EDV only, results were indistinguishable from condition (1) water only; therefore, detailed data for this condition are not shown. Analysis focused on the comparison between conditions (1) and (2).

The lineal energy values were calculated by converting oscilloscope signals using previously calibrated Geant4-based models. Entrance region measurements were calibrated at 2.0cm and 5.0cm for 70 MeV and 190 MeV protons, respectively. The same water-equivalent depths were used for actual entrance measurements, while Bragg peak measurements were taken at 3.8cm and 22.5cm for 70 MeV and 190 MeV, respectively, corresponding to distal regions of the BP with high $\bar{y_{D}}$ values.

For the 70 MeV proton beam, no statistically significant difference in $\bar{y_{D}}$ was observed between the water-only and BPA-containing conditions at either depth. A slight increase was noted near the Bragg peak but remained within the standard deviation. In contrast, for the 190 MeV beam, a statistically significant increase in $\bar{y_{D}}$ was observed at the Bragg peak in the presence of BPA (p < 0.01), while the entrance region showed no meaningful change. Two independent experimental runs were conducted for validation, and consistent results were obtained. **Figure 5** displays the data from the second set.

**Figure 6:**
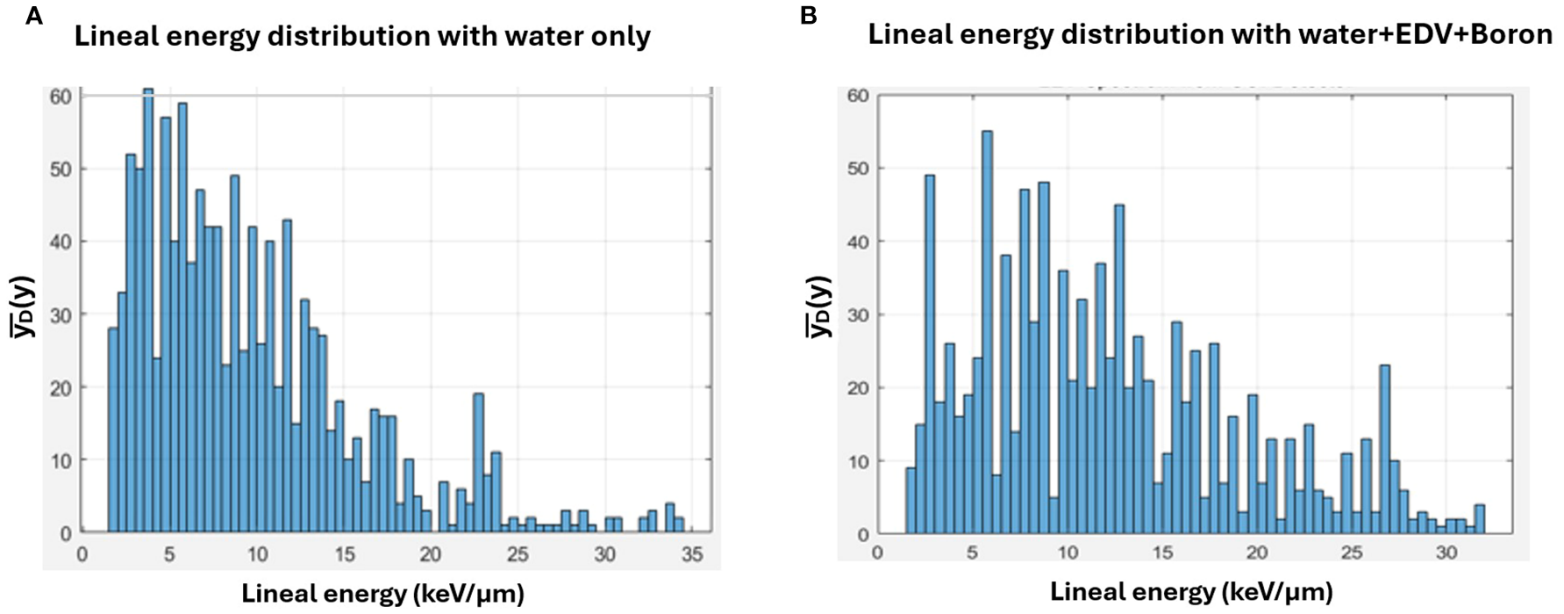
Lineal energy distributions measured at the Bragg peak region with 190 MeV protons (at depth of 22.5cm): **(A)** water only and **(B)** water + EDV + Boron (100 ppm). The dose-averaged lineal energy ( $\bar{y_{D}}$ ) was approximately 8.60 keV/μm and 11.14 keV/μm for the water-only **(A)** and boron conditions **(B)**, respectively, based on this representative measurement.

**Figure 6** illustrates representative lineal energy distributions measured at the Bragg peak for the 190 MeV beam. The y-axis represents the frequency of detected events, while the x-axis corresponds to lineal energy values in keV/μm. These distributions were used to calculate $\bar{y_{D}}$ . In the BPA-loaded case, an evident increase in high $\bar{y_{D}}$ events is evident, consistent with the theoretical expectation from the p + ^11^B → 3α reaction. To provide a more quantitative comparison of the microdosimetric spectra shown in **Figure 6**, the $\bar{y_{D}}$ was calculated for both conditions using standard microdosimetric weighting. For this representative measurement, the $\bar{y_{D}}$ was approximately 8.60 keV/μm for the water-only case (a) and 11.14 keV/μm for the boron-containing case (b). This result quantitatively supports the visual observation of increased high-lineal energy events in the presence of boron, consistent with the hypothesized proton–boron interaction effect. Although this example reflects a single measurement, the trend was reproducible across repeated experiments.

**Table 2** provides a concise numerical summary of the measured (averaged) $\bar{y_{D}}$ values for all tested conditions, enabling direct comparison between proton energies, depths, and boron presence. $\bar{y_{D}}$ values increased substantially from entrance to Bragg peak for both proton energies, reflecting the expected slowing-down effect. Boron introduction produced a measurable $\bar{y_{D}}$ enhancement at the Bragg peak for both energies, with the relative increase more pronounced at 190 MeV (from 7.88 keV/µm to 10.41 keV/µm) than at 70 MeV (from 8.02 keV/µm to 8.88 keV/µm). At the entrance depth, boron did not produce a significant $\bar{y_{D}}$ change for either energy. Notably, in the absence of boron, the standard deviations were smaller for 190 MeV than for 70 MeV, indicating improved statistical precision due to higher proton fluence. However, with boron present, the standard deviations increased for both energies, particularly at 190 MeV, likely reflecting greater variability in event energy deposition due to the additional alpha particle contributions from the proton–boron reaction.

**Table 2:** Summary of measured $\bar{y_{D}}$ values with associated standard deviations for both proton energies (70 MeV and 190 MeV) at entrance and Bragg peak depths, with and without boron.

Energy (MeV)	Depth (cm)	Boron	$\bar{y_{D}}$ (keV/µm)	Std. Dev. (keV/µm)
70	Entrance (2.0)	Without	2.14	0.56
		With	2.08	0.64
	Bragg Peak (3.8)	Without	8.02	0.81
		With	8.88	0.84
190	Entrance (5.0)	Without	2.13	0.35
		With	2.11	0.66
	Bragg Peak (22.5)	Without	7.88	0.75
		With	10.41	1.35

Each value represents the mean of ten repeated measurements under the same experimental conditions.

### 3.3 Dosimetric properties with conventional and high dose rate proton irradiation with boron density

This treatment planning study aimed to evaluate the potential impact of localized boron accumulation on biological dose distribution in proton therapy, particularly in the context of fixed versus variable RBE modelling and conventional versus FLASH dose-rate delivery. Although the macroscopic dose enhancement from proton–boron interactions is minimal, it has been hypothesized that LET modulation by localized boron could improve biological dose conformity. This simulation explores that hypothesis using a controlled treatment planning environment.

The dose calculation was performed on a liver case selected for its relatively homogeneous tissue composition and clinically relevant target size. Within the clinical target volume (CTV), a spot-array pattern (3mm diameter per spot) was delineated and assigned a physical density of 2.34 g/cm³ and a relative stopping power (RSP) of 1.18. This high-density insert does not correspond to a true 100 ppm boron concentration; rather, it served as an artificial construct to examine whether a dense material—mimicking localized boron or similar agents—could perturb dose distributions in treatment planning. The intention was to assess the physical and biological dose response in the treatment planning system (TPS), not to represent a physiologically realistic boron accumulation.

Treatment planning was performed using the Eclipse TPS (version 15.6, Varian Medical Systems), with two fields (RT and PA) optimized using single-field optimization (SFO). Fixed RBE dose distributions (RBE=1.1) were calculated using the Proton Convolution Superposition (PCS) algorithm. Variable RBE distributions were computed using the MicroCalculation script (version 1.5), which incorporates LET-based biological dose modelling. FLASH conditions were simulated by increasing the minimum MU per spot to 200, producing estimated instantaneous dose rates above 40 Gy/s based on system characteristics. Conventional dose-rate plans used a standard 2 MU per spot setting.

**Figure 7:**
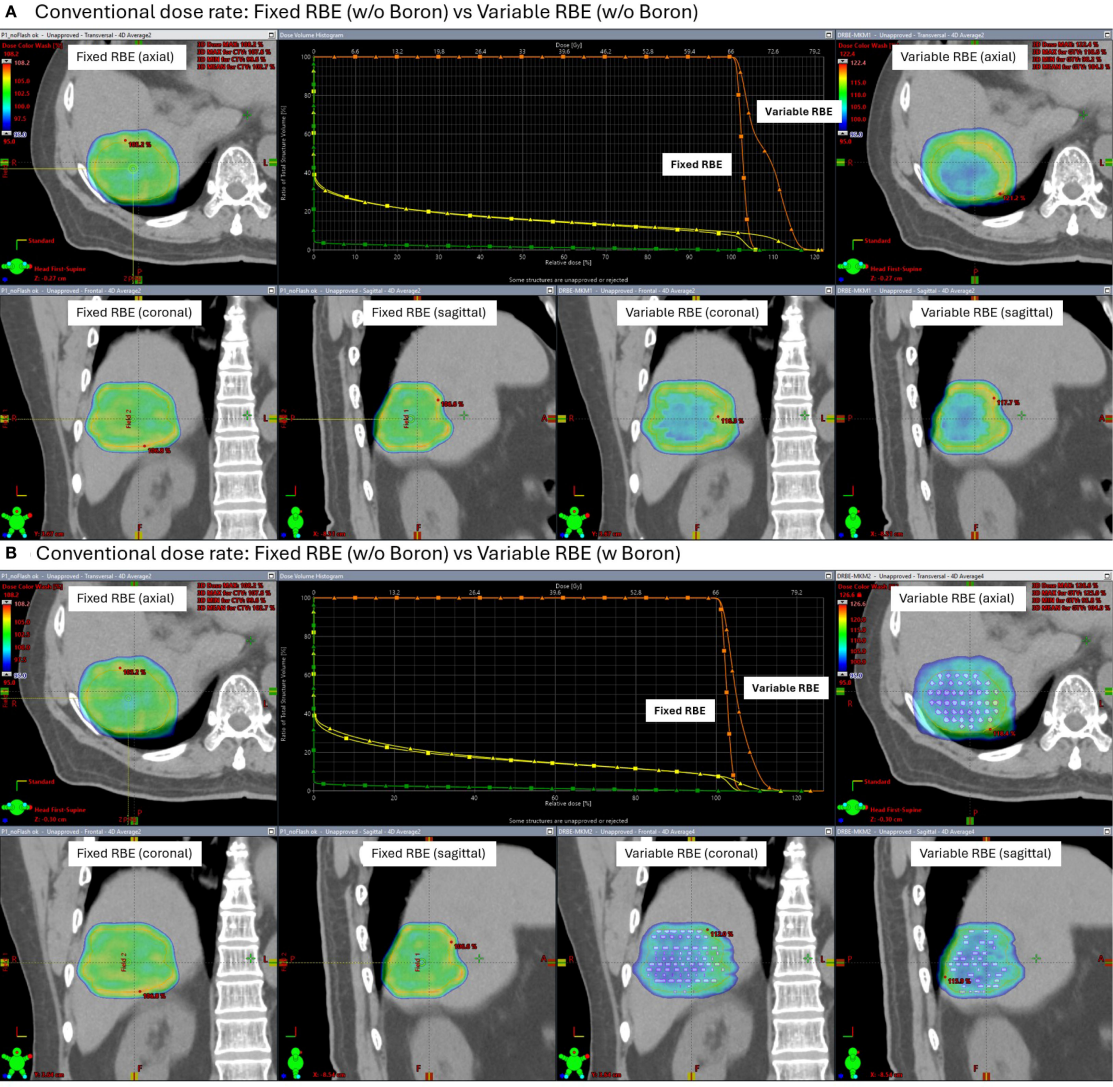
Simulated dose distributions and Dose Volume Histogram (DVH) under conventional proton therapy. Comparisons are shown between fixed and variable relative biological effectiveness (RBE) models, both without **(A)** and with **(B)** a high-density boron insert (2.34 g/cm³) placed in the CTV.

**Figure 8:**
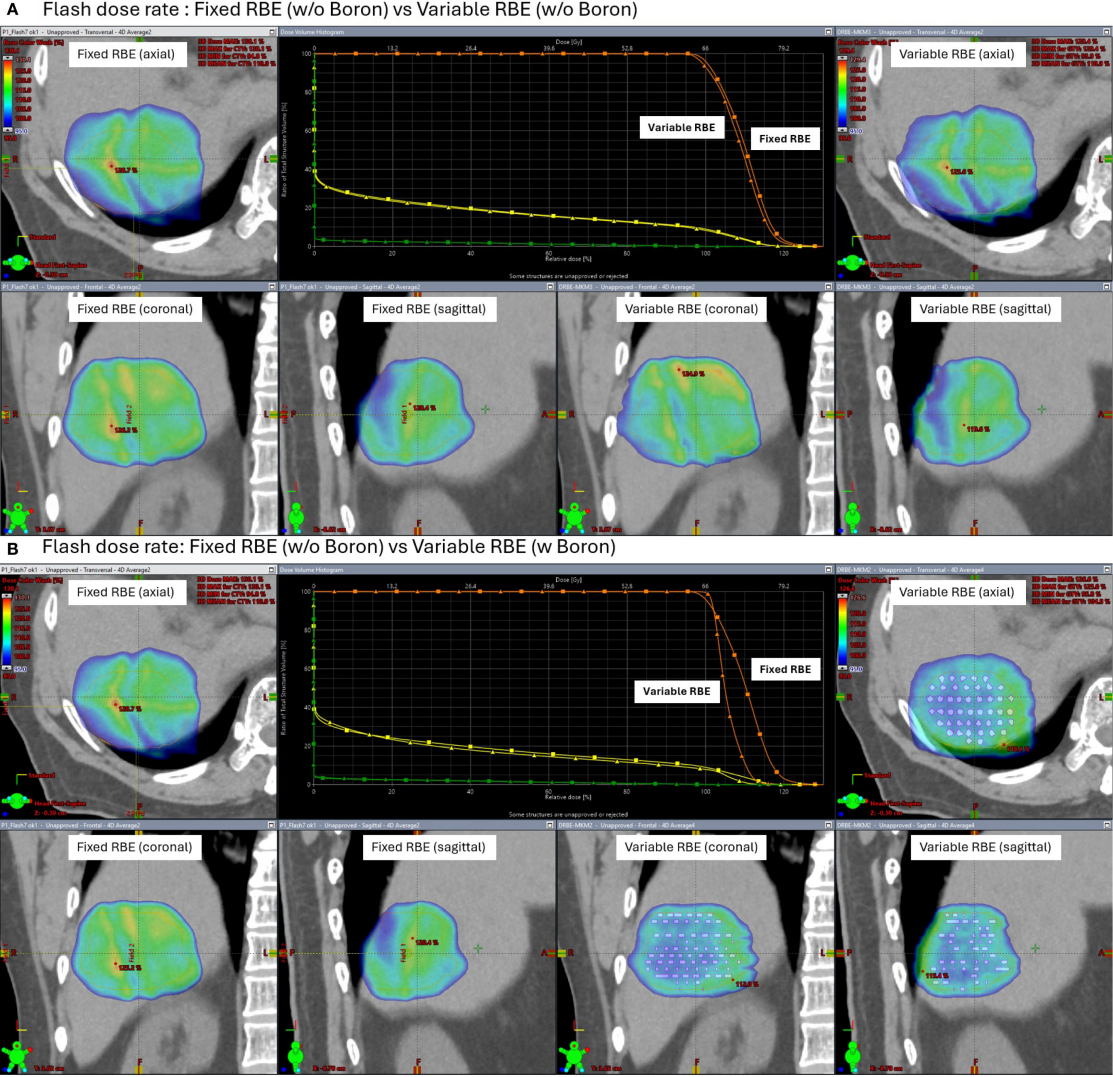
Simulated axial dose distributions and DVH under FLASH proton therapy (200 MU/spot). Comparisons are shown between fixed RBE and variable RBE models, both without **(A)** and with **(B)** a high-density boron insert in the clinical target volume (CTV). FLASH conditions reduce fixed-variable RBE differences, and the high-density boron insert region further improves biological dose uniformity.

**Figures 7** and 8 show the dose distributions and dose volume histogram (DVH) across the CTV under conventional and FLASH dose-rate conditions, respectively. Each figure includes comparisons between fixed and variable RBE dose calculations, with and without the high-density insert. Under conventional dose rates (**Figure 7**), the variable RBE plan without the insert exhibited dose heterogeneity due to LET variation. When the high-density insert was included, dose uniformity in the CTV improved, indicating that localized changes in stopping power can affect LET-weighted biological dose.

Under FLASH dose-rate conditions (**Figure 8**), the difference between fixed and variable RBE distributions was reduced even without the insert suggesting that FLASH delivery may inherently mitigate LET-driven dose variations. When the high-density region was added under FLASH, further improvement in dose homogeneity was observed, reinforcing the idea that both dose rate and local stopping power changes can influence biological dose shaping.

This study is simulation-based and does not include experimental validation. Future work will investigate these findings experimentally using phantoms embedded with high-density materials to represent localized modifiers of proton stopping power.

## 4 Discussions

SOI microdosimeter was used for measuring lineal energy with proton spot scanning irradiation system. Direct experimental comparisons with conventional microdosimeters such as TEPCs are limited due to fundamental differences in their design and application scope. TEPCs simulate tissue-equivalent energy deposition and are well suited for reference measurements in uniform or monoenergetic beams, but they have limited spatial resolution and are not optimal for high-flux or dynamically scanned beams. In contrast, SOI microdosimeter offer high spatial and temporal resolution, are resilient to pile-up, and are more suitable for scanning proton beam applications like PBS. As such, direct one-to-one comparisons between SOI and TEPC are not always feasible or meaningful. Ref. 12 highlights microdosimetric effects observed in BNCT using TEPCs with ^10^B, while Ref. 17 used SOI microdosimeter and found no significant RBE increase under their experimental PBCT/NCEPT conditions. Our study supports $\bar{y_{D}}$ enhancement from proton–^11^B interactions, consistent with the PBCT mechanism, which may contribute to increased RBE, even if not directly to macroscopic dose enhancement.

A key difference between previous studies on lineal energy measurements using SOI detectors (16) and our investigation lies in the signal processing method. In prior studies, measurements were performed using a multi-channel analyzer (MCA). In our study, a linear model-based conversion method was used to derive lineal energy distributions directly from oscilloscope signals, without an MCA. To address signal fluctuations, averaged data analysis was performed post-irradiation. Additionally, we investigated the effect of boron on lineal energy during proton irradiation in the context of proton boron capture therapy (PBCT).

To ensure alpha particles could reach the sensitive volume (SV) of the SOI microdosimeter, we modified the original PMMA sheath configuration. The default wall thickness between the boron-containing well and the SV was 0.5 mm, which is too thick for alpha particles—having sub-50 μm ranges in water—to penetrate. Therefore, the phantom wall was intentionally broken and replaced with a custom-made barrier using two layers of wrapping plastic. This wrapping material, identified as low-density polyethylene (LDPE, density 0.92 g/cm^³^), had a total physical thickness of approximately 40 μm. Given the density difference from PMMA (1.18 g/cm^³^), the resulting water-equivalent thickness was estimated at 37 μm, which is comparable to ∼31 μm of PMMA. This modification allowed alpha particles generated from the proton–boron reactions to potentially reach the SV, supporting the validity of observed LET changes. This structure is the basis for the 30–50 μm thickness range mentioned in the method.

The linear calibration coefficients “a” and “b” in Equation 1 were obtained via least-squares regression at the entrance calibration depth for each proton energy. The standard errors from the fit indicated relative uncertainties of U_a_/a is 2–4% and an absolute intercept uncertainty of U_b_ is 0.05–0.3 keV/µm (1σ). Propagation of these uncertainties to $\bar{y_{D}}$ showed that the calibration process contributed an estimated ±3% (1σ) uncertainty to $\bar{y_{D}}$ . Main sources of uncertainty include counting statistics in the calibration spectra, oscilloscope noise and baseline drift, detector gain variations, and small mismatches between the Monte Carlo calibration geometry and the physical detector setup.

It is important to clarify that our experimental setup used natural BPA containing approximately 80% ^11^B and 20% ^10^B, without any isotope enrichment. Therefore, the observed $\bar{y_{D}}$ enhancement is unlikely to be due to neutron capture effects from ^10^B, and should not be interpreted within the context of NCEPT (neutron capture enhanced particle therapy), because alpha particle energy from 10B(n.alpha)7Li reaction is about 1.5 MeV and will be stopped in 30-40 μm water equivalent layer. The observed differences are attributed to proton interactions with ^11^B nuclei.

The study by Cirrone et al. (3) investigated PBCT by treating human prostate cancer DU145 cells with sodium borocaptate (BSH) and irradiating them with a clinical proton beam. Their results demonstrated a significant increase in cell killing and chromosomal aberrations in the presence of boron, particularly at the distal end of the spread-out Bragg peak (SOBP), where proton energies are lower and the probability of the p + ^11^B → 3α reaction is higher. The resulting high-LET alpha particles were believed to contribute to enhanced biological damage. However, several other reports (6–8) have presented conflicting or inconclusive findings regarding PBCT efficacy.

In our study, we specifically focused on demonstrating $\bar{y_{D}}$ increases during proton irradiation with boron by using natural boronophenylalanine (BPA), containing approximately 80% ^11^B and 20% ^10^B. Proton energies of 70 MeV and 190 MeV were selected for the pristine Bragg peak measurements in a solid water phantom. This selection was made to align with previous studies: the lower energy (70 MeV) similar to Ref (3)., and the higher energy (190 MeV) matching that used in Ref (11). We also emphasize that our experiments were performed using monoenergetic, single-energy spot beams (70 MeV and 190 MeV), unlike Ref (3)., which used a spread-out Bragg peak (SOBP) delivery. In a SOBP, the energy spectrum at the cell or detector location is broader and typically includes more lower-energy components, which could increase the reaction probability and $\bar{y_{D}}$ variation. Thus, direct quantitative comparison between SOBP-based biological findings and our monoenergetic measurements is limited, and this distinction may explain some of the differences in reported outcomes.

As shown in **Figure 4**, our measurement method, after calibration against Geant4 simulation results, demonstrated reasonable $\bar{y_{D}}$ as a function of depth. Based on this calibration, we proceeded to assess the boron effect on $\bar{y_{D}}$ . In the comparison between boron-loaded and non-boron conditions (**Figure 4**), all experimental parameters (phantom setup, irradiation conditions, and signal calibration/conversion) were kept identical, ensuring valid comparisons.

Although $\bar{y_{D}}$ can increase at lower proton energies due to slowing-down effects, in our measurements the $\bar{y_{D}}$ values ranged from approximately 2–8 keV/μm for both the 70 MeV and 190 MeV beams (**Figure 4**). Notably, the relative $\bar{y_{D}}$ enhancement with boron was more clearly observed at 190 MeV than at 70 MeV (**Figure 5**). This difference cannot be attributed to dose or nominal $\bar{y_{D}}$ alone, as these were matched across conditions. A likely explanation is the substantially higher proton fluence delivered at 190 MeV, resulting from higher transmission efficiency and optimized cyclotron settings. For the same number of monitor units, the 190 MeV beam delivered more than three times the proton fluence of the 70 MeV beam (**Table 1**), which in turn improved statistical sampling, increased event counts, and reduced relative uncertainty in the $\bar{y_{D}}$ estimation. This higher statistical quality makes subtle boron-induced shifts in $\bar{y_{D}}$ more detectable.

By contrast, the lower fluence at 70 MeV may have limited the sensitivity to small changes, even though the cross-section for the p + ^11^B → 3α reaction increases with decreasing proton energy. This fluence discrepancy is an inherent limitation of the current work. Measurements were performed in QA mode at the Bragg peak using fixed monoenergetic beams, and fluence control for Bragg-peak-only delivery is not straightforward at lower energies on the Varian system. In clinical practice, however, Varian systems employ Spread-Out Bragg Peaks (SOBP). Future studies will therefore use TPS-based SOBP simulations and measurements to better replicate clinical conditions and enable fluence-matched comparisons across different beam energies.

Mazzone et al. (24) reported there is no significant dose increasement with Proton Boron interaction. While our experimental $\bar{y_{D}}$ increases appear more pronounced, we suggest that this could be due to microdosimetric sensitivity of the SOI microdosimeter to localized alpha particle ionization events near the sensitive volume. The reason we select energy of 70 MeV and 190 MeV is to investigate previous cell experiments results from Ref [3, 4, and 11].

Although macroscopic dose enhancement from proton–boron capture is known to be very low due to the rare occurrence of p + ^11^B → 3α reactions, an increase in $\bar{y_{D}}$ —even without a measurable dose increase—can still elevate biological effectiveness. $\bar{y_{D}}$ reflects energy deposition density on a microscopic scale, which is more relevant for predicting cellular damage than dose alone. Thus, the observed $\bar{y_{D}}$ increase may partly explain the enhanced biological effects seen *in vitro*, despite the absence of substantial dose amplification.

In this study, we observed that the uncertainties in the $\bar{y_{D}}$ measurements were relatively larger in the entrance region for both 70 MeV and 190 MeV proton beams as shown in **Figure 4**. This increased variability is primarily attributed to the lower energy deposition and reduced signal amplitude in the entrance region, where the proton $\bar{y_{D}}$ is inherently low. The limited signal-to-noise ratio under these conditions contributes to higher measurement fluctuation. While we repeated each measurement ten times to improve statistical reliability, the smaller absolute energy transfer in the entrance region makes the system more sensitive to minor fluctuations. Future work with larger sample sizes, extended acquisition times, or enhanced detector sensitivity could help to further reduce this uncertainty. Such improvements may allow more definitive assessment of potential boron-induced effects in regions where subtle changes in $\bar{y_{D}}$ could otherwise remain undetectable. Despite these limitations, the current data provide reproducible and statistically significant $\bar{y_{D}}$ enhancement in the Bragg peak region, which remains the primary focus for potential PBCT benefit.

Although BPA dissolved in water was used in the experimental setup to reflect a clinically relevant boron carrier, the treatment planning simulations employed a simplified model with a localized, high-density boron region. This approach was selected to explore the upper-bound dosimetric effect of boron under idealized conditions. A more realistic simulation of dissolved BPA was not feasible within the current limitations of the Eclipse treatment planning system, which does not support voxel-wise modelling of heterogeneous elemental distributions or time-dependent uptake patterns. This localized, high-density approach allows us to evaluate whether boron—if concentrated in the target region—could produce a meaningful change in physical dose metrics such as homogeneity or LET. It should be noted that the Eclipse TPS does not support voxel-wise simulation of dissolved agents or heterogeneous chemical distributions like BPA. As such, a simplified geometric model was used within the limitations of the TPS to mimic the presence of boron.

As a result, the localized boron model used here should be regarded as an exploratory scenario rather than a direct clinical representation. Future work will focus on incorporating voxelized boron distributions derived from experimental biodistribution data, such as PET imaging or autoradiography, and integrating pharmacokinetically informed uptake models. Such developments would allow simulation of spatially heterogeneous boron concentrations and their temporal evolution, thereby improving the clinical relevance of PBCT treatment planning studies.

For FLASH condition study, two beam configuration was set in TPS (conventional and FLASH-style), also two different dose calculation models are used with fixed RBE (1.1) and a variable RBE model (MicroCalculation). The FLASH condition was emulated by creating a dedicated beam model in the TPS with minimum MU per spot set to 200 MU, resulting in shorter delivery times per field. The purpose of this approach was not to evaluate FLASH-specific biological effects or to validate the accuracy of dose-rate modelling, but rather to explore whether introducing such high-MU delivery constraints would still produce consistent and acceptable dosimetric distributions within the TPS simulations, without implying experimental validation. The MicroCalculation model adjusts biological dose based on LET distributions but does not account for dose-rate or temporal effects. Therefore, this planning scenario serves to test the feasibility and consistency of LET-weighted biological dose distributions under modified delivery conditions, rather than to assess robustness against a reference biological model. It should be noted that the FLASH-related findings in this study are limited to TPS-based simulations. The high-MU/spot delivery emulated in the planning system ensured dose-rate values above 40 Gy/s, but the model does not incorporate radiobiological FLASH mechanisms such as oxygen depletion or radical quenching. Therefore, the FLASH scenarios presented here should be interpreted only as feasibility tests of LET-sensitive RBE modelling under altered delivery conditions, not as evidence of biological FLASH effects in PBCT. Additionally, the use of a localized high-density boron region may alter the water equivalency and marginally reduce proton range. Even a shift of ~0.5 mm could position the SOI microdosimeter further into the distal Bragg peak, where $\bar{y_{D}}$ (bar is needed) is strongly depth-sensitive, particularly for high-energy protons with greater straggling. Although our TPS confirmed no change in absorbed dose distribution, the microdosimetric spectrum is dose-independent, and this effect could have contributed to the observed $\bar{y_{D}}$ (bar is needed) enhancement. Future experiments with improved phantom manufacturing tolerances and range verification will be important to resolve this issue. 

## 5 Conclusion

This study investigated the potential of PBCT by measuring lineal energy using a SOI microdosimeter under clinically realistic and theoretical conditions. Our experiments demonstrated a measurable increase in $\bar{y_{D}}$ at the Bragg peak region when boron was introduced, particularly at 190 MeV proton energy. This enhancement, although modest, aligns with previous cell-based studies suggesting increased biological effectiveness in PBCT. The observed energy-dependent difference in boron-induced $\bar{y_{D}}$ enhancement was likely influenced by the substantially higher proton fluence at 190 MeV compared to 70 MeV, which improved statistical sensitivity to subtle effects.

While the overall dose enhancement due to proton–boron interactions is expected to be minimal due to the low cross-section of the p + ^11^B → 3α reaction, the localized increase in $\bar{y_{D}}$ may still contribute to biological effects not captured by conventional dosimetry.

Additionally, we performed treatment planning simulations comparing conventional and FLASH-style delivery using fixed and variable RBE models. While these simulations did not account for explicit realistic boron distribution conditions, the resulting biological dose distributions underscore the importance of LET-weighted planning in high-dose-rate scenarios.

Taken together, our findings support further exploration of PBCT as a complementary strategy in proton therapy, especially in combination with LET-aware treatment planning. Future work should involve refined modelling of boron pharmacokinetics, external validation with other microdosimetric detectors, and integration of dose-rate effects to more accurately assess the synergy between boron delivery and FLASH proton irradiation.

## Data Availability

The raw data supporting the conclusions of this article will be made available by the authors, without undue reservation.
